# Evaluation of Liver Transplantation Outcomes Using Grafts From Brain‐Dead Donors With Warfarin‐Associated Coagulopathy

**DOI:** 10.1155/joot/8367205

**Published:** 2026-05-30

**Authors:** Behrouz Seydi Majd, Mohammad Mehdi Lashkarizadeh, Alireza Shamsaeefar, Saman Nikeghbalian, Hamid Zaferani Arani, Sahar Sohrabi Nazari, Seyyed Mohammad Sadegh Ahmadi Rashti, Ahmad Khalid Sanaei, Hamed Nikoupour

**Affiliations:** ^1^ Shiraz Transplant Center, Abu-Ali Sina Hospital, Shiraz University of Medical Sciences, Shiraz, Iran, sums.ac.ir

**Keywords:** brain-dead donors, extended-criteria donors, liver transplantation, marginal donors, warfarin-associated coagulopathy

## Abstract

**Background:**

Liver transplantation is the definitive therapy for end‐stage liver disease. The severe organ shortage necessitates exploring grafts from extended‐criteria donors, including those who become brain dead due to toxicological causes. This study evaluated the outcomes of liver transplantation using grafts from brain‐dead donors with confirmed warfarin‐associated coagulopathy (WAC).

**Methods:**

This retrospective observational cohort study was conducted at a high‐volume transplant center (1994–2024). Ten recipients who received liver grafts from brain‐dead donors with WAC were included. Clinical data were reviewed to assess liver function recovery, complications, and survival.

**Results:**

The cohort included 10 recipients (8 males, 2 females; mean age: 49.8 ± 13.2 years). Liver function tests and international normalized ratio (INR) levels showed significant improvement by Postoperative day 7. Acute rejection occurred in two recipients (20%), successfully treated with corticosteroids. One patient (10%) developed primary nonfunction, leading to death. Patient survival was 100% at 30 days and 70% at 1 year; corresponding graft survival was 90% and 70%.

**Conclusion:**

In this small, highly selected cohort, liver grafts from brain‐dead donors with WAC may be used with acceptable short‐term safety. These preliminary findings demonstrate feasibility and highlight the need for larger, controlled studies to further evaluate outcomes and donor selection criteria.

## 1. Introduction

Liver transplantation is the definitive therapy for end‐stage liver disease (ESLD) and selected liver malignancies [1]. The severe global shortage of donor organs necessitates the responsible use of grafts from extended‐criteria donors (ECDs) [2, 3]. Donors who have suffered brain death due to drug overdose represent one such potential source [4].

Among these, donors with warfarin‐associated coagulopathy (WAC) present a unique challenge. Warfarin, a vitamin K antagonist, induces a pharmacological coagulopathy characterized by a markedly elevated international normalized ratio (INR) [5]. While fatal intracranial hemorrhage from warfarin overdose can lead to brain death suitable for donation, the associated coagulopathy has historically raised concerns about intrinsic hepatic dysfunction and graft suitability [5, 6]. However, this coagulopathy results from the inhibition of clotting factor synthesis rather than parenchymal damage and is potentially reversible posttransplantation given warfarin’s half‐life [5, 6].

Despite this pathophysiological rationale, robust clinical data on the safety and outcomes of liver transplantation using grafts from donors with confirmed WAC are scarce. Therefore, this retrospective observational cohort study aimed to systematically evaluate the clinical outcomes of liver transplantation using grafts from brain‐dead donors with WAC at a high‐volume transplant center.

## 2. Materials and Methods

### 2.1. Study Design and Population

**FIGURE 1:**
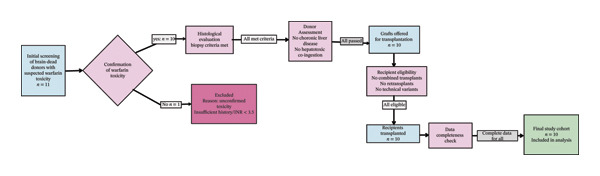
CONSORT‐style flowchart of donor and recipient selection.

This retrospective observational cohort study was conducted at the Abu‐Ali Sina Organ Transplantation Center in Shiraz, Iran. We included all adult patients (≥ 18 years) who underwent primary, standard whole‐liver transplantation between 1 January 1994 and 31 December 2024 using grafts from brain‐dead donors with confirmed WAC. The screening and inclusion process is detailed in Figure 1.

### 2.2. Inclusion and Exclusion Criteria

Donor eligibility required documented evidence of warfarin ingestion leading to brain death. WAC was confirmed by either an INR > 3.5 at any point during the clinical course or a clear history of overdose with an INR > 1.5 deemed the primary cause of fatal intracranial hemorrhage. All alternative causes of coagulopathy were excluded. A mandatory pretransplant biopsy confirmed preserved hepatic architecture (< 20% macrovesicular steatosis, Ishak fibrosis score ≤ 2, no necroinflammatory activity).

Recipients were included if complete pretransplant data and at least 12 months of postoperative follow‐up were available, except in cases of early graft failure. We excluded recipients of combined organ transplants, retransplants, or technical variant grafts, as well as cases with incomplete records, evidence of preexisting donor liver disease, or loss to follow‐up.

### 2.3. Recipient Selection and Ethical Considerations

Grafts were reviewed by a multidisciplinary transplant committee. Recipients were selected based on high clinical urgency (e.g., Model for ESLD [MELD] score > 25) or risk of waitlist mortality. Explicit informed consent, detailing the marginal nature of the graft and the specific risks associated with WAC, was obtained from all recipients. The study was approved by the Institutional Review Board (IR.SUMS.MED.REC.1404.050). The requirement for individual patient consent for the retrospective analysis was waived.

### 2.4. Data Collection and Outcomes

Data were collected from electronic and paper records. Primary endpoints were patient and graft survival at 30 days and 1 year, and early graft function (INR ≤ 1.5 by Postoperative day 7 [POD7]). Secondary endpoints included acute cellular rejection (Banff 2016 criteria), primary nonfunction (PNF), major adverse events (Clavien–Dindo grade ≥ III), infections, and renal replacement therapy. PNF was defined as death or the need for retransplantation within 7 days, accompanied by an AST ≥ 3000 U/L and at least one additional criterion (INR ≥ 2.5, arterial pH < 7.3, or lactate ≥ 4 mmol/L).

### 2.5. Postoperative Management

A standardized protocol included intravenous vitamin K and fresh frozen plasma to correct coagulopathy, and a triple‐drug immunosuppressive regimen.

### 2.6. Statistical Analysis

Data were analyzed using SPSS Version 21 (IBM Corp., Armonk, NY). Given the small sample size (*n* = 10), the analysis was exclusively descriptive. Continuous variables are presented as mean ± standard deviation and categorical variables as frequencies.

## 3. Results

### 3.1. Baseline Characteristics

From an initial screening of 11 potential cases, we included 10 liver transplant recipients (8 males and 2 females) with a mean age of 49.8 ± 13.2 years. Recipient demographics and clinical characteristics are detailed in Table 1. The mean MELD score was 18.5 ± 4.2.

**TABLE 1:** Baseline characteristics of liver transplant recipients (*n* = 10).

Variable	Value (mean ± SD or *n* (%))	Range
Age (years)	49.8 ± 13.2	18–65
Sex (male:female)	8:2	—
BMI (kg/m^2^)	26.5 ± 3.8	18.5–32.1
MELD score	18.5 ± 4.2	12–25
Child–Pugh class (A/B/C)	3 (30)/5 (50)/2 (20)	—
INR	1.8 ± 0.3	0.8–2.5
Total bilirubin (mg/dL)	5.2 ± 2.1	0.3–15
AST (U/L)	95 ± 40	20–300
ALT (U/L)	78 ± 35	10–250
Etiology		
‐ HCV	3 (30)	
‐ NASH	2 (20)	
‐ PSC	2 (20)	
‐ HBV	1 (10)	
‐ Other^∗^	2 (20)	

*Note:* AST = aspartate aminotransferase, HCV = hepatitis C, NASH = nonalcoholic steatohepatitis, HBV = hepatitis B, ALT = alanine aminotransferase.

Abbreviations: BMI = body mass index, INR = international normalized ratio, MELD = Model for End‐stage Liver Disease, PSC = primary sclerosing cholangitis.

^∗^Secondary biliary cirrhosis and intrahepatic cholangiocarcinoma.

The mean donor age was 32.4 ± 8.6 years. All donors had a documented history of warfarin overdose with an initial INR > 3.5. The mean INR at organ procurement was 2.23 ± 1.08, reflecting partial correction after medical management. Pretransplant biopsies showed preserved histoarchitecture with < 5% macrovesicular steatosis.

### 3.2. Intraoperative Data

The piggyback technique was used in 80% of cases. Biliary reconstruction was duct‐to‐duct in 70% and Roux‐en‐Y hepaticojejunostomy in 30%. Mean cold ischemia time was 420 ± 100 min, warm ischemia time was 40 ± 10 min, and intraoperative blood loss was 1500 ± 300 mL. Transfusion requirements included 4.1 ± 2.1 units of packed red blood cells and 3.5 ± 2.3 units of fresh frozen plasma.

## 4. Postoperative Outcomes

### 4.1. Liver Function Recovery

**FIGURE 2:**
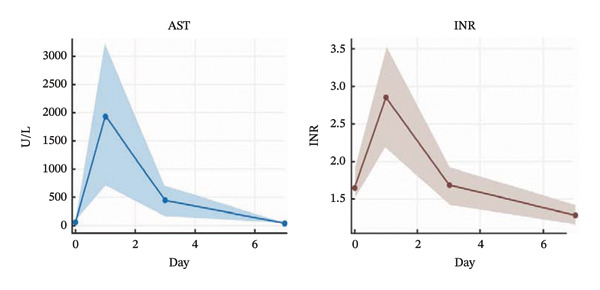
Trends in postoperative liver function recovery.

INR normalized from 2.85 ± 1.07 preoperatively to 1.29 ± 0.2 by POD7. Aspartate aminotransferase decreased from 1950.8 ± 2007.8 U/L to 53.2 ± 20.6 U/L (Figure 2).

### 4.2. Complications

Acute rejection occurred in two patients (20%), successfully treated with steroids. One patient (10%) developed PNF. Major adverse events (Clavien–Dindo ≥ III) occurred in four patients (40%): two required renal replacement therapy, one had severe sepsis, and one had ischemic‐type biliary cholangiopathy.

### 4.3. Survival

Patient survival was 100% at 30 days and 70% at 1 year. Graft survival was 90% and 70%, respectively. The three deaths within one year were due to PNF (*n* = 1), COVID‐19 pneumonia (*n* = 1), and recurrent cholangiocarcinoma (*n* = 1).

## 5. Discussion

This small, retrospective observational cohort study provides preliminary evidence that liver allografts from carefully selected brain‐dead donors with WAC can be transplanted with acceptable short‐term safety in a highly screened population. The absence of a control group precludes any conclusion regarding equivalence to standard grafts, but our findings support the technical feasibility of this approach.

The rapid normalization of recipient INR by POD7 aligns with warfarin’s pharmacokinetics, confirming that the coagulopathy was pharmacologically mediated rather than indicative of intrinsic hepatic dysfunction [5, 7]. This is consistent with literature on trauma‐associated coagulopathy, where elevated INR does not preclude successful transplantation when hepatic histology is preserved [8, 9].

The single case of PNF occurred in a recipient who experienced the longest cold ischemia time and significant intraoperative hemodynamic instability. This implicates ischemia‐reperfusion injury rather than donor warfarin exposure as the primary cause, reinforcing that graft viability in WAC donors depends on the absence of structural liver damage and minimization of perioperative insults [10].

Our intraoperative and postoperative outcomes, including blood loss, transfusion requirements, and a 20% rejection rate, fell within expected ranges for liver transplantation at our center [11–13]. This suggests that using grafts from donors with WAC does not introduce unique technical or immunogenic risks in this controlled setting.

However, these results must be interpreted within significant limitations. The very small sample size, single‐center design, and highly selective inclusion criteria (e.g., mandatory biopsy and exclusion of steatotic grafts) limit generalizability and likely select for an optimal donor subgroup. The extended study period (1994–2024) introduces temporal heterogeneity in surgical and medical management. Most critically, the lack of a matched control group prevents direct comparison with recipients of standard criteria grafts.

Given these limitations, our findings are hypothesis‐generating. They suggest that a protocol‐driven evaluation of WAC donors may be justified. Key steps should include (1) documentation of warfarin overdose and INR trajectory, (2) mandatory pretransplant biopsy to rule out parenchymal injury, and (3) transplantation into carefully selected recipients after thorough informed consent.

## 6. Conclusion

In this small, highly selected cohort, liver transplantation using grafts from brain‐dead donors with WAC appears feasible with acceptable short‐term outcomes. These preliminary data argue against the automatic discard of such organs but highlight the need for larger, multicenter, comparative studies to robustly quantify risks, refine selection criteria, and establish evidence‐based guidelines for their use.

## Funding

No funding was received for this study.

## Conflicts of Interest

The authors declare no conflicts of interest.

## Data Availability

The data that support the findings of this study are available on request from the corresponding author. The data are not publicly available due to privacy or ethical restrictions.
